# No Reef Is an Island: Integrating Coral Reef Connectivity Data into the Design of Regional-Scale Marine Protected Area Networks

**DOI:** 10.1371/journal.pone.0144199

**Published:** 2015-12-07

**Authors:** Steven R. Schill, George T. Raber, Jason J. Roberts, Eric A. Treml, Jorge Brenner, Patrick N. Halpin

**Affiliations:** 1 Caribbean Program, The Nature Conservancy, Coral Gables, Florida, United States of America; 2 Department of Geography and Geology, The University of Southern Mississippi, Hattiesburg, Mississippi, United States of America; 3 Marine Geospatial Ecology Lab, Nicholas School of the Environment, Duke University, Durham, North Carolina, United States of America; 4 School of BioSciences, University of Melbourne, Melbourne, Victoria, Australia; 5 Texas Chapter, The Nature Conservancy, Houston, Texas, United States of America; Biodiversity Research Center, Academia Sinica, TAIWAN

## Abstract

We integrated coral reef connectivity data for the Caribbean and Gulf of Mexico into a conservation decision-making framework for designing a regional scale marine protected area (MPA) network that provides insight into ecological and political contexts. We used an ocean circulation model and regional coral reef data to simulate eight spawning events from 2008–2011, applying a maximum 30-day pelagic larval duration and 20% mortality rate. Coral larval dispersal patterns were analyzed between coral reefs across jurisdictional marine zones to identify spatial relationships between larval sources and destinations within countries and territories across the region. We applied our results in Marxan, a conservation planning software tool, to identify a regional coral reef MPA network design that meets conservation goals, minimizes underlying threats, and maintains coral reef connectivity. Our results suggest that approximately 77% of coral reefs identified as having a high regional connectivity value are not included in the existing MPA network. This research is unique because we quantify and report coral larval connectivity data by marine ecoregions and Exclusive Economic Zones (EZZ) and use this information to identify gaps in the current Caribbean-wide MPA network by integrating asymmetric connectivity information in Marxan to design a regional MPA network that includes important reef network connections. The identification of important reef connectivity metrics guides the selection of priority conservation areas and supports resilience at the whole system level into the future.

## Introduction

The rapid decline in coral reef health [1,2,3,4] is prompting countries around the world to take actions to increase coral reef conservation and management. Marine Protected Areas (MPAs) are one of the most widely advocated methods for protecting coral reefs [5], and many countries and regions are seeking to expand protection of coral reef habitat [6,7]. To stay healthy, coral reefs rely heavily on ocean currents that provide new recruits from near and far locations [8,9]. These demographic linkages are a key ecological support system for coral reefs, and previous research suggests that reef connectivity has a strong influence on community-level biomass, population persistence, resilience, and species diversity [10]. However, these currents do not follow political boundaries and several studies suggest MPA networks rarely achieve their full potential because connectivity is typically not incorporated into a regional design process [11,12,13,14,15]. Consequently, a key challenge in the MPA network design process is to identify the appropriate size, spacing, and location of MPAs in order to safeguard sufficient connectivity processes that will maintain a healthy functioning ecosystem while acting as a mutually replenishing network to facilitate the recovery of populations following a disturbance [10,16,17].

Clearly, more research is needed to find cost effective and meaningful pathways for incorporating ecological connectivity into MPA design [18]. One of the main problems is in identifying the scale of marine larval dispersal, a fundamental challenge at the intersection of marine ecology and oceanography disciplines [19,20,21]. Several studies suggest that confronting the coral reef crisis is going to require regional collaboration and scaling-up of management efforts that focus on improving our understanding of the ecological processes that underlie reef resilience [22,23,24]. Accordingly, countries need to work together to understand and protect patterns in coral larval dispersal and collaboratively design strategic system-wide MPA networks across multiple marine jurisdictions [12,25,26].

To address these challenges, we modeled larval dispersal across coral reefs in the Caribbean and Gulf of Mexico to identify important reef connections on a regional scale. Questions driving our research were: Following a spawning event, where do coral larvae go? Where is settlement and recruitment most likely to occur? How dependent are reefs within each jurisdiction on recruits from each local or upstream reef in other jurisdictions? Where are the key source sites of marine connectivity within the region and are they protected? In order to answer these questions, we modeled coral population connectivity based on a 30-day maximum larval dispersal period across eight spawning events from 2008–2011 using a spatially explicit connectivity model [25,26]. We used this information in the conservation planning software Marxan to identify a suite of coral reef priority areas that meet conservation targets while maintaining important connections between reef populations. Building on previous Marxan marine connectivity studies [27,28,29], our research provides additional insight because: the analysis represents a synthesis of data over four years including multiple spawning events; 3) we quantify and report larval connectivity data by Exclusive Economic Zones (EZZ); and 4) we use the connectivity information in a systematic conservation planning program to design a regional MPA network that includes important reef connections. By identifying important shared reef connections between marine jurisdictions, we hope to promote multilateral cooperation in coral reef protection and management, maintaining highly-connected populations which could aid in disturbance recovery and improve reef resilience [26].

## Methods

We modeled coral connectivity and integrated the results into a conservation optimization algorithm to identify priority reef conservation areas within ten marine ecoregions that make up the Caribbean Basin, Gulf of Mexico and the southwest Sargasso Sea (8–35 N, 56–98 W) [30]. Several studies have investigated the ecological connectivity of this region [19,31,32,33,34], however our work is unique in that it integrates connectivity data into a conservation decision-making framework, providing insight for both ecological and political contexts.

### Coral Larvae Dispersal Model

#### Reef Data

**Fig 1:**
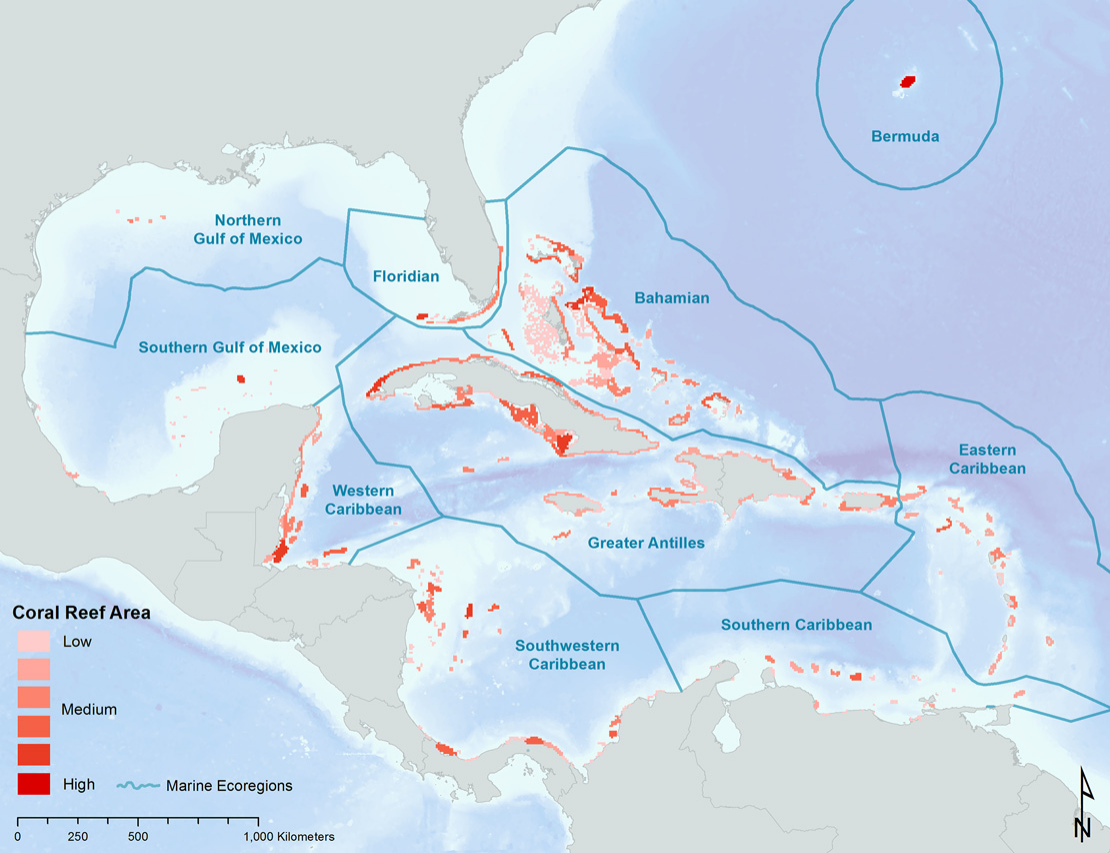
General reference map showing the location of the ten marine ecoregions used in the analysis indicating the distribution of total reef area within each of the 423 reef units that were used in the larvae transport and settlement simulation. Coral reef data used are from the Millennium Coral Reef Mapping Project [35] which represents the most accurate and consistently mapped global distribution of shallow coral reef systems.

We used coral reef data from the Millennium Coral Reef Mapping Project [35] as a consistent and high-resolution representation of coral reef locations throughout the Caribbean Basin and Gulf of Mexico. Prior to using these data, all coral reef locations were reviewed and edited by in-country reef experts. We developed a gridded reef map (8x8 km) and grouped contiguous clusters of coral habitat into 423 distinct reef units (Fig 1). Given the close proximity of the coral reefs within each reef unit, it was assumed that each unit was internally connected.

#### Ocean Current Data

Ocean current data were acquired from the NOAA Real-Time Ocean Forecast System (RTOFS) database [36]. The RTOFS database distributes daily ocean current data integrating tidal patterns and is based on the Hybrid Coordinate Ocean Model (HYCOM) [37]. The model is a basin-scale ocean forecast system for the northern Atlantic and part of the southern Atlantic Ocean, using a variable size grid resolution ranging from 4 to 17 km, and extending from 25°S to 72°N and to 98°W to 16°E. Evaluations of the RTOFS performance indicate that the modelled ocean data compare well to historical observations at regional scales [38].

#### Dispersal Model

We modeled the dispersal of reef building coral larvae for spawning events using a spatially-explicit larval dispersal model [38,39,40]. We modelled two simulations per year from 2008 to 2011 with each simulation starting on the last quarter moons of August and September, based on observations of coral mass spawning events [41,42,43]. These dates were 23 August 2008, 22 September 2008, 13 August 2009, 12 September 2009, 1 September 2010, 1 October 2010, 21 August 2011, and 20 September 2011.

The dispersal model included the following parameters to quantify connectivity: time and frequency of spawning, pelagic larval duration, settlement behaviour, and larval mortality (**Table 1).** In each simulation, the amount of larvae released was proportional to the reef area within each reef unit. For each simulation, we used a maximum pelagic larval duration (PLD) of 30 days [24,31,44] and a mortality rate of 20% day^-1^. Although the influence of mortality is well recognized [45,46], field-based data is extremely limited. A recent review [45] and laboratory data on several corals [47,48] suggest mortality is often variable in time (often higher earlier in the larval duration period) and within a cohort, but generally on the order of 5% day^-1^ to 10% day^-1^, with some corals experiencing up to approximately 35% day^-1^ mortality [48]. We modelled a full range of mortality rates, but use the 20% day^-1^ rate for illustration purposes.

**Table 1:** Descriptions and values of coral larval biological parameters used in the dispersal simulations.

Larval Biological Parameter	Description	Value
**Time and frequency of spawning (*e*.*g*. lunar, annual)**	This defines the larval release times in the model. More spawning opportunities have significant implications on the local-to-regional connectivity patterns.	We performed eight dispersal simulations—two per year—that started on the dates of the last quarter moon -based on observations of coral mass spawning events in the Caribbean (23 August 2008, 22 September 2008, 13 August 2009, 12 September 2009, 1 September 2010, 1 October 2010, 21 August 2011, 20 September 2011).
**Maximum pelagic larval duration (PLD)**	The PLD representing the maximum amount of time larvae can spend in the water column.	We used a maximum PLD of 30 days.
**Pre-competency period**	The period of early development when larvae are not capable of settlement. This is often between 2–7 days for many invertebrates.	Larval competency was modeled using a gamma cumulative distribution function [38] that allowed all of the larvae to reach full competency in 3 days [39,48].
**Settlement behaviour**	Probability of larvae settling if they encounter a suitable habitat cell.	After reaching competency, when larvae are over coral habitat they settled at a rate of 75% per day.
**Local density and fecundity**	Represents the relative reproductive output from individual reef patches.	The amount of larvae released was proportional to the amount of reef area per habitat patch.
**Larval mortality**	This daily mortality rate of larvae while dispersing.	At each daily time-step (24 hours) during the simulation a mortality factor of 20% was applied to the amount of settled larvae for that time step.
**Migration rate threshold to determine ‘meaningful’ connectivity**	This limit, in terms of settlement likelihood, provides a way of distinguishing between ecologically relevant connectivity. See [39] for a more in-depth discussion and considerations with respect to reproductive output.	We used 1/1,000,000 larvae as a cut-off for ecologically relevant connectivity.

**Fig 2:**
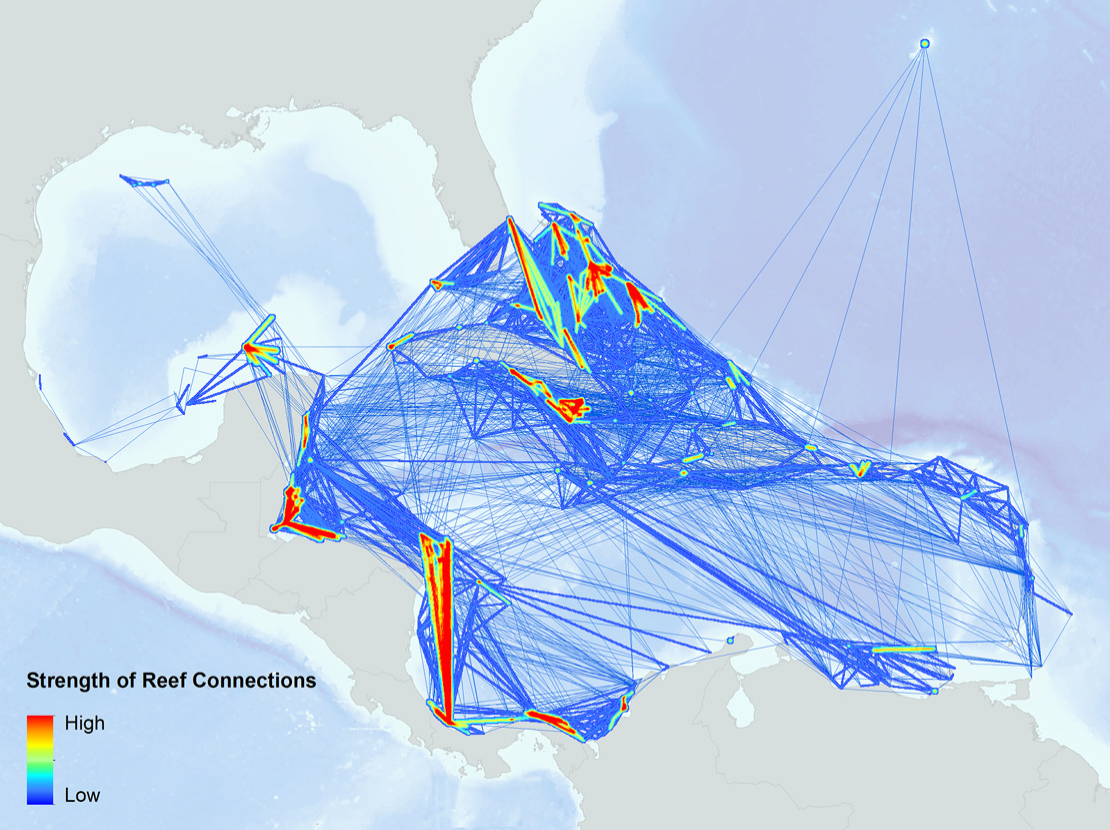
Strength of reef connections based on modeled transported coral larvae. These values represent an average of eight coral larvae dispersal simulations between 2008–2011. The width and color of the lines represent the strength of connection. The darker red and orange areas indicate high amounts of settled coral larvae transported along that connection, while the shades of blue represent smaller amounts of settled larvae.

The primary output of each simulation represented an estimate of the total amount of larvae transported between each of the 423 reef units, including local-retention. We calculated a time-averaged connection strength by averaging the total settled larvae across simulations and the probability of larval dispersal among all reefs. Dispersal networks were used to visualize these connection strengths among all possible source-destination reef pairs (Fig 2).

### Connectivity Assessment

#### Centrality Measures

We used a centrality measure to represent conservation value. In our connectivity network, we transformed the edge weights using *x*–*connection strength*, where *x* is next whole number greater than the largest connection strength value. In this way, connection strength is the same rank-order as geographic distance, a prerequisite of these centrality measures. Similar to White et al [27] and Holstein et al [31], we calculated *betweenness* and *closeness* centrality measures using values in our reef network. The Python package *NetworkX* was used for the network analysis. *Betweenness* centrality is calculated by determining the number of times a particular node (i.e., reef patch) serves as a stepping-stone in the shortest paths between all other pairs of nodes in the network. This measure can be used to identify important stepping-stones that facilitate connectivity in a network. *Closeness* centrality values are higher for a particular node when its total distance to all other nodes in the network is lower. This measure indicates how close a particular node is to the other nodes in the network.

#### MPA Network Design: Marxan Overview

We used Marxan (v. 2.42) [49,50], a widely adopted conservation planning software, for selecting marine conservation priority areas when considering coral larval dispersal connections between reef units across the Caribbean Basin and Gulf of Mexico. Marxan selects a set of planning units that best minimize predefined costs, while attempting to meet certain user defined conservation targets. An example target may be the desire to include 20% of all reef area within the set of priority areas, or more generally, 20% of a conservation feature whatever it may be (e.g. a particular habitat, species). The primary result of Marxan is a set of priority areas which are selected as a balance of the user-defined targets and the underlying costs. Results provide decision-makers with a portfolio of sites that can be evaluated for inclusion in a conservation area network design [11,27,51,52].

Targets are set by the user for each conservation feature under consideration for inclusion in the resulting set of priority areas. Marxan has several other inputs (some of which are optional) that inform the algorithm about the cost of creating a potential set of priority areas from an input set of planning unit sites [49]. Using the “objective function,” Marxan evaluates a potential set of conservation priority areas based on a score of the sites or planning units that are selected for inclusion in the set of priority areas. In our case, these will be the reef planning units. The general form of this function is:

$$\sum_{sites}^{}Cost+BLM\;\sum_{sites}Boundary\;+\;\sum_{conValue}\;CFPF\;\times Penalty+Cost\;Threshold\;Penalty\;\left(t\right)$$

[49]

The *Cost* represents the sum of the costs associated with the planning units or sites that will comprise the selected conservation priority area. These can be the actual cost associated with acquiring the area, or opportunity costs, or some entirely different metric of cost as defined by the user.

The *Boundary* represents the actual length of the boundary of the selected priority area. Using the actual length of the boundary allows Marxan to prioritize solutions that create reserve systems with smaller external boundaries. This has the effect of creating clumped priority areas which may be desirable in certain situations (e.g. potential for easier designation and management). Costs are sometimes alternatively used as “boundaries.” In this research we demonstrate the use of the strength of the connection between reefs as a boundary cost between reef units. In this way, as Marxan seeks to find potential priority areas by minimizing the external boundary, it clumps the priority areas based on the strength of the connections between reef units. In other words, a reef unit with a strong connection will be more likely to be selected than a disconnected reef, especially if has similar amounts of other costs and contributes similarly to meeting the user defined targets.

The *Penalty*, often referred to as the “conservation feature penalty factor” is a term that allows the user to express a cost associated with a set of priority areas that do not meet the established targets. Increasing this value will cause Marxan to increase the total cost of the solution in the case where a target is not met for a set of priority areas. Thus, increasing a conservation feature’s penalty makes it more likely Marxan will select an output solution that will meet the target for that conservation feature. Finally, the *Cost Penalty Threshold* is a cost associated with exceeding the total user-defined cost for the potential set of priority areas. For this study, we applied a cost penalty value between 1.0–1.4 which allowed the algorithm to find a reasonably efficient solution.

Selecting a set of conservation priority areas is iterative and Marxan offers several algorithms that can be selected from to determine the process whereby the objective function is implemented. The factors in the objective function are affected by the makeup of the initially selected set, and then recalculated for the current selected priority areas for each iteration. Therefore, the solution is complex and the algorithm works towards a final solution that meets user-defined conservation targets while minimizing cost ─represented by *t* in the formula above. In practice, Marxan is run a number of times, each with different initial solution which may produce different final results. The primary output in this case, is the “best” solution over all the runs, which is the one that meet the targets and captures the lowest overall cost (including the penalty factors). Another commonly used output is the “sum of solutions” which will report the number of times each planning unit or site was included in the set of priority areas.

#### Marxan-based Conservation Scenarios

In order to explore the differences between a typical Marxan analysis and one which includes connectivity data, we compare the results of Marxan using two scenarios: a) a typical scenario where conservation targets are set using reef area (per reef unit) and a boundary file that uses a transformation of Euclidean distance to reef units (i.e. nearby reefs effectively shared a larger common boundary); and b) a connectivity-based scenario where conservation targets are set using local retention and betweenness centrality (per reef unit) and a boundary file that uses the asymmetric strength of connection values calculated between reef units. We chose to use Euclidean distance in the first scenario because reef units rarely shared an actual boundary, and we needed a surrogate for boundary length in order to clump model results and provide a manageable output solution. To test the influence of using a boundary length modifier, a third scenario with a single 30% target of reef area was tested, but it didn’t use a boundary file. The results appeared to be driven solely by the reef units with the largest reef area and lowest cost, since Marxan was simply balancing the selection of reef units by reef area and cost.

We evaluated and compared results of the two scenarios using two stratification schemes: a) a single region strata (*i*.*e*. where targets can be met anywhere within the study area); and b) a multiple region stratification using the ten marine ecoregions (*i*.*e*. where targets are met within each marine ecoregion). For all scenarios, we set a 30% target and penalty factor of 10 for the conservation feature(s) being considered in each scenario and ran 100 repetitions with one million iterations each using the simulated annealing algorithm with iterative improvement, commonly used by Marxan users [53]. The decision to use a 30% target and a penalty factor of 10 is illustrative. We experimented with other values that resulted in more or less areas being selected, but 30% seemed like an appropriate balance for demonstrating our research purpose. When running Marxan, it is helpful to visualize the results using different target values as an exploratory measure so that one can better understand the tradeoffs between various inputs values [51]. For scenario “a,” setting a target of 30% for the single conservation feature of reef area meant that Marxan would be seeking to select at least 30% of the reef area within the study area. Failure to do so would result in a penalty factor of 10. In scenario “b,” we used the betweeness centrality and local retention values that had been assigned to each reef unit and assigned the target of 30%. In other words, we desired that Marxan include in its solution 30% of all the betweeness centrality values by reef unit (favoring the highly connected reefs) and 30% of all local retention values by reef unit (favoring large self-sustaining reefs). A Boundary Length Modifier (BLM) value of 0.17 was used for both scenarios after calibrating by analyzing boundary length and cost relationships for multiple runs at various BLM values. Planning unit cost per reef unit was derived by taking the average value from a 1 km cell cumulative global marine threat model developed by Halpern et al [54].

#### Selection of Connectivity Features

For the connectivity-based Marxan scenario, we set a 30% targets for a) local retention of larvae; and b) betweenness centrality of reef units. Local retention of larvae is a measure of the proportion of larvae that were released that remain in the natal patch. Higher values of local retention suggest that a reef is more likely to be self-sustaining [55]. Upon evaluation of the centrality measures, we determined that betweenness centrality was best suited in the Marxan analysis because it identifies important reefs that are important pathways within the network.

#### Selection of Boundary Length

The Boundary Length Modifier (BLM) parameter is a multiplicative factor in the Marxan algorithm, which attempts to minimize the boundary length to area ratio, thus increasing the continuity of the solution set. High BLM values force the clustering of the solution set, whereas low BLM values allow for a more fragmented set to be selected as a model solution. Typically, boundary length often is represented by the measured length of the boundary between each pair of planning units that share a common border. In the Marxan selection algorithm, removing planning units that share a large common boundary will incur a greater cost than removing those that have a smaller common boundary or no boundary at all. In our analysis, we used the reef units as the planning units, however, these units did not share any actual common boundaries as they exist apart from each other. In the reef area target scenario, the boundary length was based on Euclidian distance between reef units. However, since Marxan expects connected planning units to have larger values, we first scaled all the distance pairs to a range of 0–1 and then reversed them to be on a scale of 1–0: a value of 1 meaning that that pair had a distance of 0 (meaning a self-connection) and 0 being the pair of reef units furthest from each other. For the connectivity-based scenario, we used the asymmetric strength of connection values calculated between reef units and a similar operation to scale connection strength values. In this scenario, it was not necessary to reverse these as the values were already in an order that descended from the strongest connections down to the lowest. In choosing a BLM value for each Marxan scenario, we calculated a calibrated value based on an analysis of boundary length and cost using multiple runs.

## Results

Our results are summarized in the following products: Animation of the larval transport models, analysis of the larval dispersal (received and contributed) and local retention by Exclusive Economic Zone (EEZ), analysis of centrality measures by reef unit, and a comparison of Marxan scenarios.

### Larval Transport Animations

**Fig 3:**
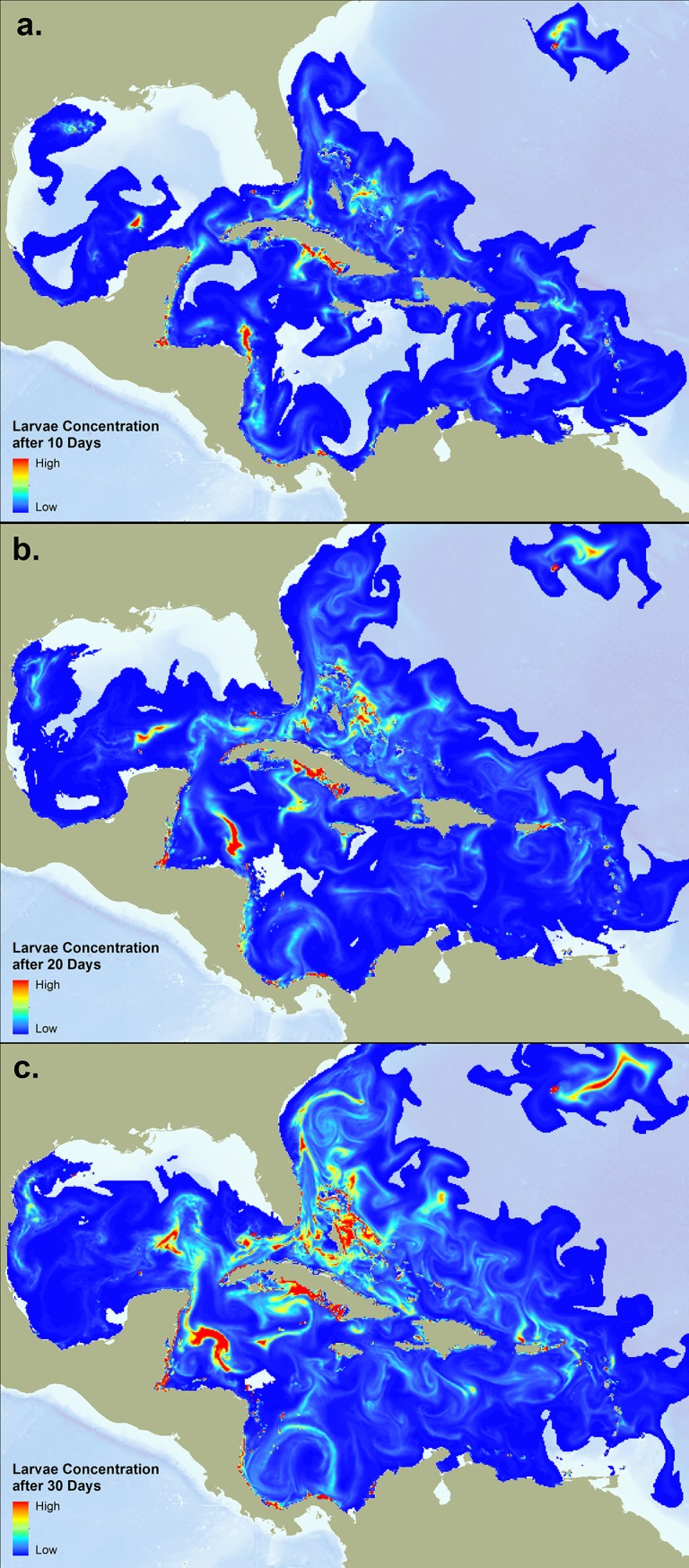
Visualization of a 30-day simulated coral spawning event based on NOAA’s Real-Time Ocean Forecast System (RTOFS) ocean current data starting on August 21, 2011. The amount of coral larvae released was based on reef area. These maps represent time steps during the 30-day pelagic larvae duration model, representing coral larvae distribution and concentration after a) 10 days; b) 20 days; and c) 30 days. These data were used to create the hourly animations for each of the eight spawning events.

Visualization of larval dispersal probabilities is a useful tool for understanding ocean dynamics and how coral reef ecosystems depend on each other. We created of a series of hourly animations showing modeled larval dispersal for each spawning event. An example of the data used to create several frames within the animation for the spawning event on August 21, 2011 can be seen in Fig 3. These animations can be accessed at the following link (http://tnc.usm.edu/connectivity/animations.zip).

### Larval Dispersal summarized by EEZ

**Fig 4:**
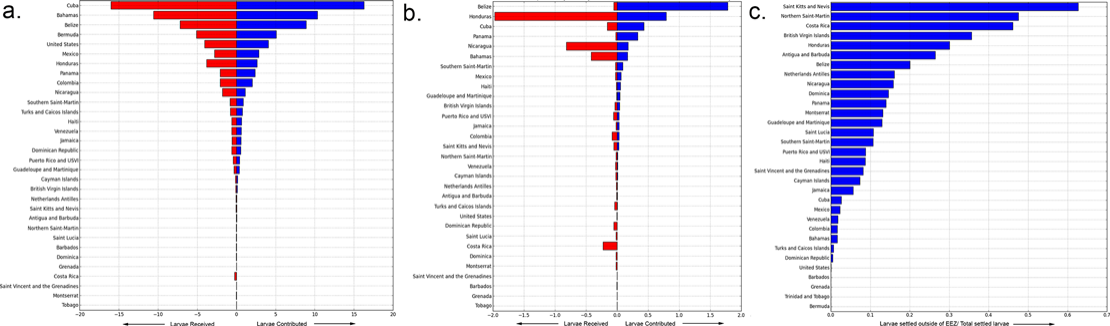
a) Modeled larvae settlement rates by EEZ averaged across eight spawning events showing total received larvae (red) and contributed larvae (blue). The red bar indicates the total modeled larvae received and settled within each corresponding EEZ. The highest amounts of larvae received and contributed are largely influenced by an EEZ’s reef area, ocean current patterns, and geographic location. Refer to the individual country maps to see these results in map format. The blue bar represents the total modeled larvae that originated within each EEZ and settled anywhere. b) The same data, but ignoring larvae that originates and settles within the same EEZ. For example, according to the model, Belize receives very little larvae that originate outside its EEZ. However, Belize contributes more larvae to other EEZs than any other EEZ. Honduras on the other hand, receives the most incoming larvae from other EEZs, while contributing the second highest level of larvae to other EEZs. c) Larvae contribution ratio by EEZ showing the proportion of all settled larvae that originates within each respective EEZ and is contributed to other EEZs.

**Fig 5:**
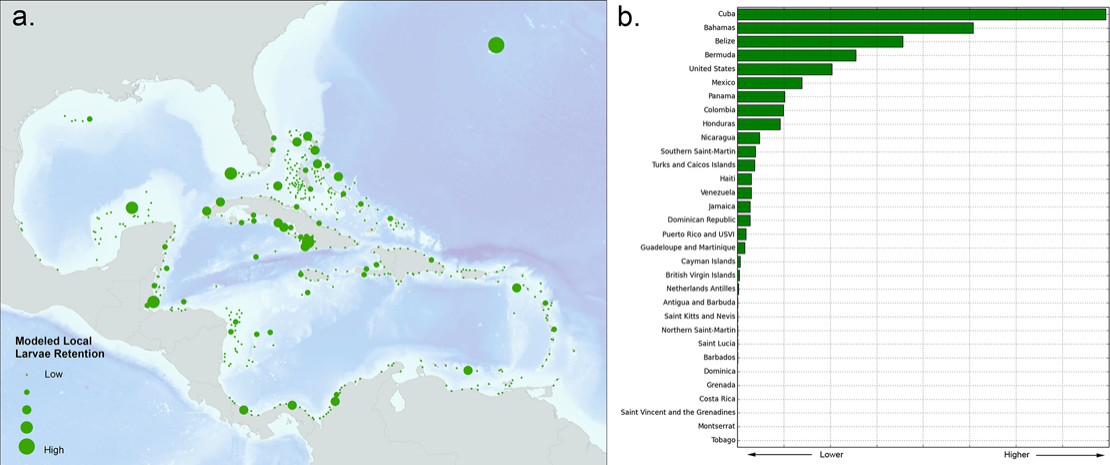
a) Modeled larvae settlement that originates and settles within the same reef unit (*i*.*e*. local -retention); b) Modeled larvae settlement of that originates and settles within the same EEZ.

**Fig 6:**
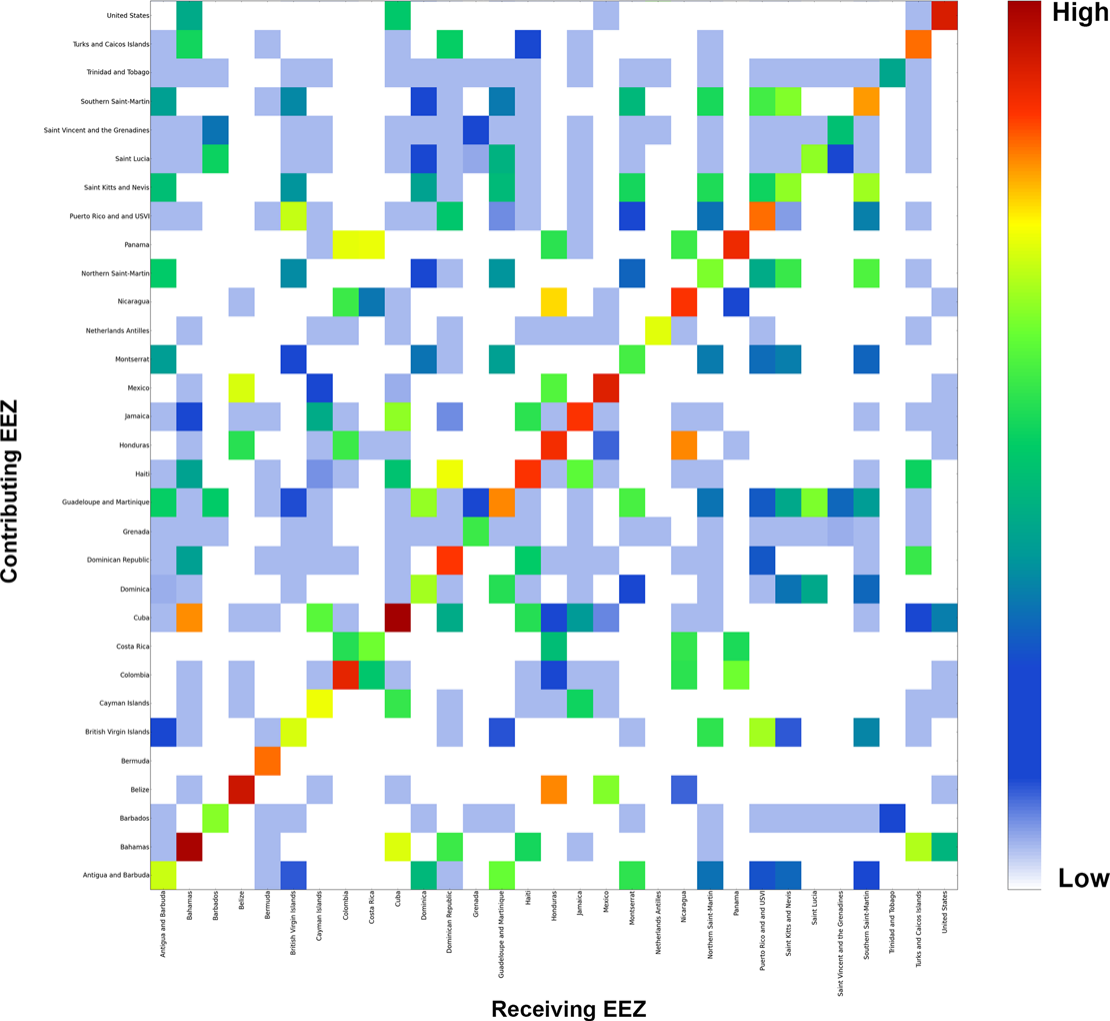
Connectivity matrix by EEZ showing the relative strength of each country connection based on the amount received (x-axis) and contributed (y-axis) settled larvae.

Using the modeled reef connection strength results, we analyzed larvae recruitment and settlement exchange rates as well as local larval retention based on 32 Exclusive Economic Zones (EEZ). Figs 4, 5 and 6 help answer questions such as: “Outside each individual marine jurisdiction, where do coral larvae come from?” (*e*.*g*. Outside of Cuba, where do Cuba’s coral larvae come from?) and “Into what jurisdictions do an individual jurisdiction’s coral larvae settle?” (*e*.*g*. In what countries do Cuba’s coral larvae settle?).

Fig 4 graphs the results of EEZ coral connectivity analysis in three ways. The first graph (Fig 4A) shows modeled coral larvae settlement rates by EEZ averaged across the eight modeled spawning events. Total larvae received are the red bars to the left (indicating the total modeled larvae the model settled within each corresponding EEZ) and contributed larvae are the blue bars to the right (representing the total modeled larvae that originated within each EEZ and settled anywhere). Cuba, Bahamas, and Belize are the top EEZs that both receive and contribute larvae to other EEZs. In the second graph (Fig 4B), the same data are visualized, except we exclude local retention (*i*.*e*. larvae that originates and settles within the same EEZ). For example, according to the model, Belize captures much of its own larvae, receiving much less larvae that originate outside its EEZ. Honduras on the other hand, receives the most incoming larvae from other EEZs, while contributing the second highest level of larvae to other EEZs. In the last graph **(** Fig 4C), we see the a ratio of contributed coral larvae by EEZ showing the proportion of all settled larvae that originates within each respective EEZ (denominator) to all settled larvae contributed to other EEZs (numerator). This is same as the blue bar in (b) divided by the blue bar in (a) for each EEZ. St. Kitts and Nevis is the only jurisdiction in which a higher percentage of originating settled larvae settled outside its own EEZ. These values should be thought of as larvae probabilities or percent incoming or outgoing, not the prediction of actual larvae numbers, and are largely influenced by an EEZ’s reef area, ocean current patterns, and geographic location.

We also evaluated local retention by reef unit, or the degree to which each reef unit is self-sustaining (larvae originates and settles in the same reef unit). Fig 5(A) shows the spatial distribution of modeled local retention rates by reef unit. Fig 5(B) is a graph of the same information, only summarized by EEZ, showing the sum of all values for connections where both the “from” and “to” reef units were within the same EEZ.

Although Figs 4 and 5 highlight important connectivity spatial patterns and ranking of summaries by EEZ, they lack the ability to provide from-to information. For example, we can see Belize contributes significant larvae amounts to other jurisdictions, but to which jurisdictions do the larvae settle? Conversely, when considering Honduras, from where do the receiving larvae come? In order to address these questions, we created a connectivity matrix that summarized all the unique combinations of from-to relationships by EEZ jurisdiction pairs using the connection strength dataset (Fig 6). This matrix shows the relative strength of each country connection based on the amount of settled larvae received (x-axis) and contributed (y-axis) from each respective EEZ. For example, when interpreting the matrix, it is apparent that much of the contributed larvae that originated in Belize are received in Honduras, with smaller amounts going to both Mexico and Nicaragua. On the other hand, the strongest external contribution that Honduras makes is to Nicaragua.

### Coral connectivity network analysis

**Fig 7:**
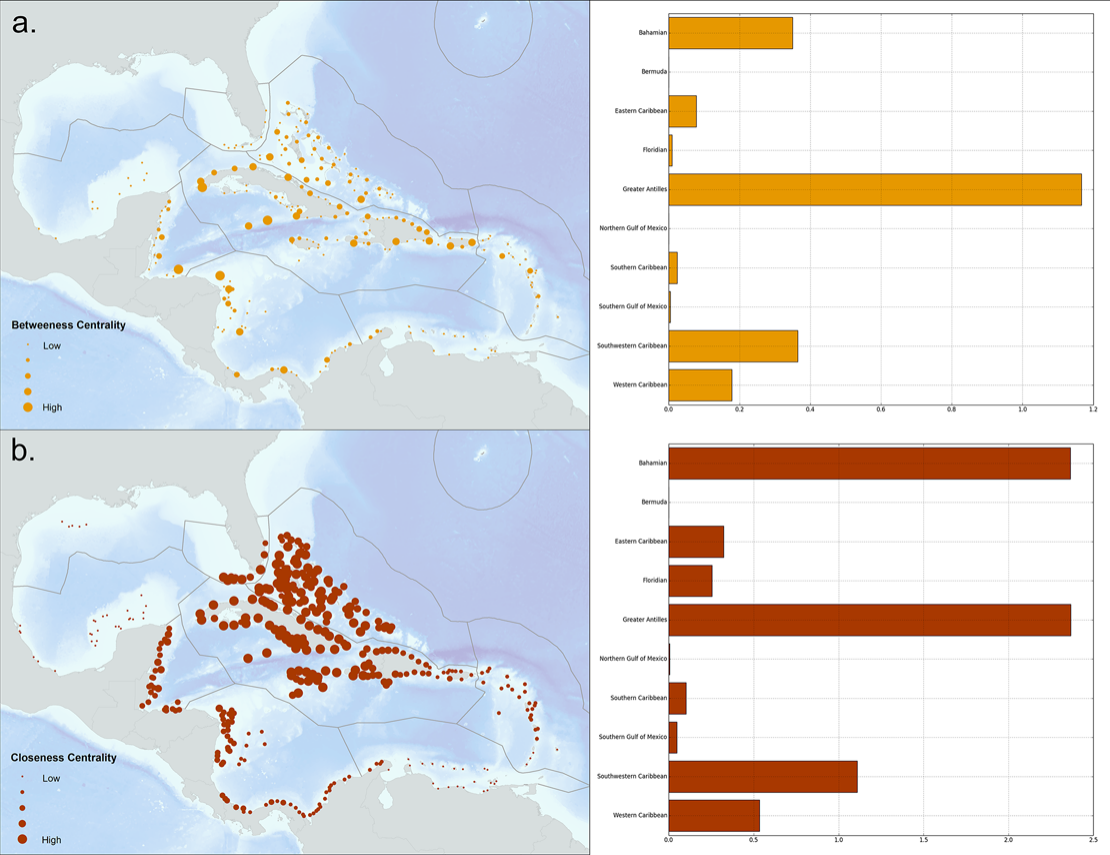
a) Betweenness centrality measures by reef unit indicating the importance of each reef unit’s role in maintaining network connectivity. The corresponding graph shows betweenness centrality measures summed by marine ecoregion; b) Closeness centrality measures by reef unit indicating how long it will take to spread something from a particular node to the other nodes in the network. The corresponding graph shows closeness centrality measures summed by marine ecoregion.

The results of each of the two centrality measures (*betweenness* (a), *closeness* (b)) calculated as part of the network graph analysis are shown in Fig 7. The sum of each of these measures by marine ecoregion appears next to the respective map for each measure. Upon interpreting these maps, it becomes apparent that *betweenness* captures many of the important bridges that maintain connectivity within the region while the other two measures characterize the core or center of the network. For this reason, and the fact that previous reef connectivity research had used this measure, we use the betweenness scores for target setting in the Marxan analysis. Based on model results, reefs on the edges of larger islands or land masses that are closest to other islands have higher betweeness centrality measures (*i*.*e*. Cuba, Hispaniola, Belize, and Honduras). Island clusters such as The Bahamas and northern side of Cuba score high in this measure due to the high density of reefs in these areas.

### Marxan results

**Fig 8:**
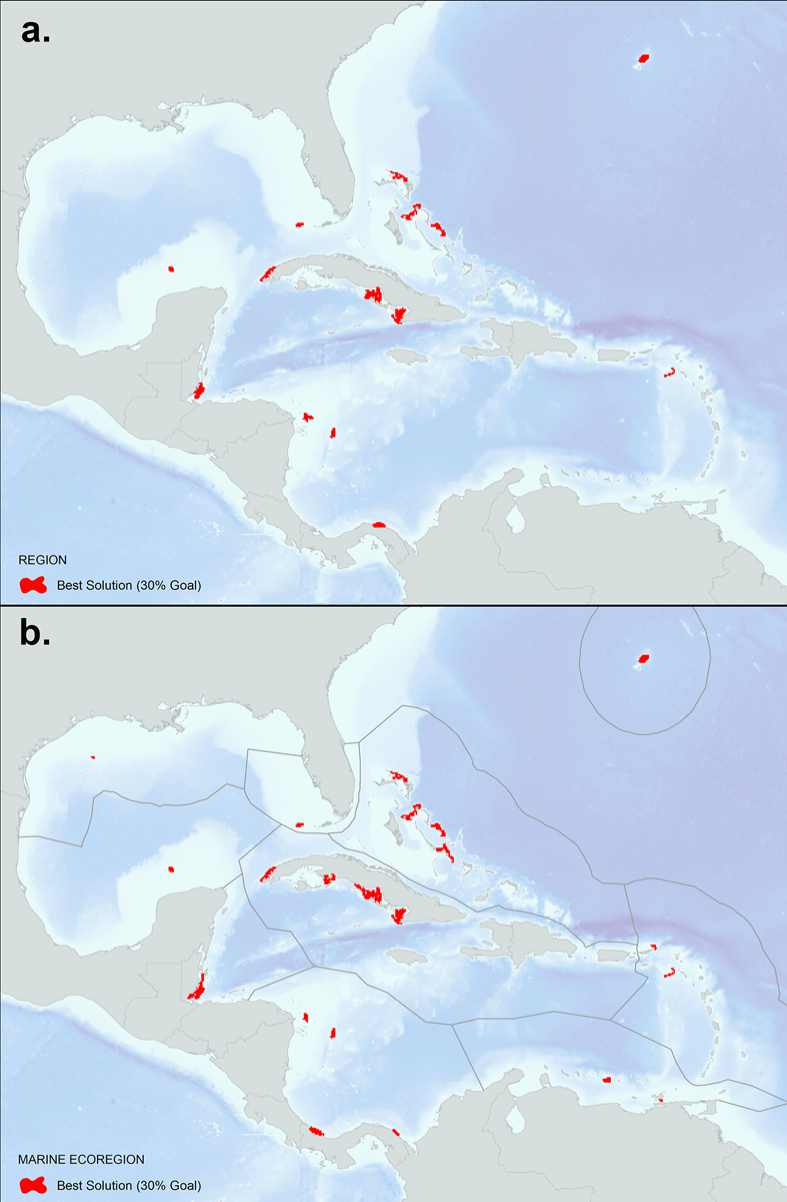
Typical Marxan best solution (not considering coral connectivity) that met a 30% target for reef area only as summarized by reef unit: a) regional assessment (no strata) and b) stratified by marine ecoregion. These results were based on 100 repetitions using a million iterations per run with a calibrated BLM value of 0.17. A penalty factor of 10 was used and a boundary file based on the Euclidean distance between reef units. The calculated cost value by reef unit was derived from the Global Map of Human Impacts to Marine Ecosystems [54].

**Fig 9:**
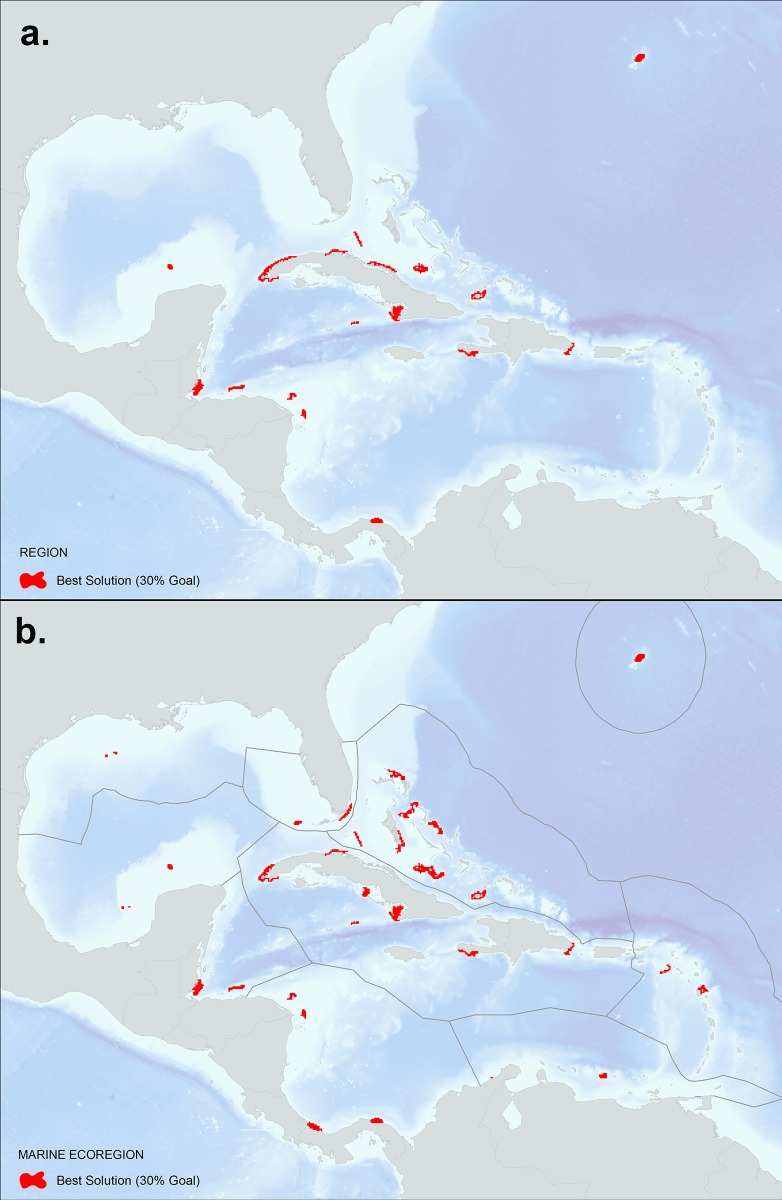
Marxan coral connectivity best solution that met a 30% target set for local retention and betweenness centrality values by reef unit: a) regional assessment (no strata); and b) stratified by marine ecoregion. These results were based on 100 repetitions using a million iterations per run with a calibrated BLM value of 0.17. A penalty factor of 10 was used and an asymmetric boundary file based on the amount of settled larvae that traveled between reef units. The calculated cost value by reef unit was derived from the Global Map of Human Impacts to Marine Ecosystems [54].

**Fig 10:**
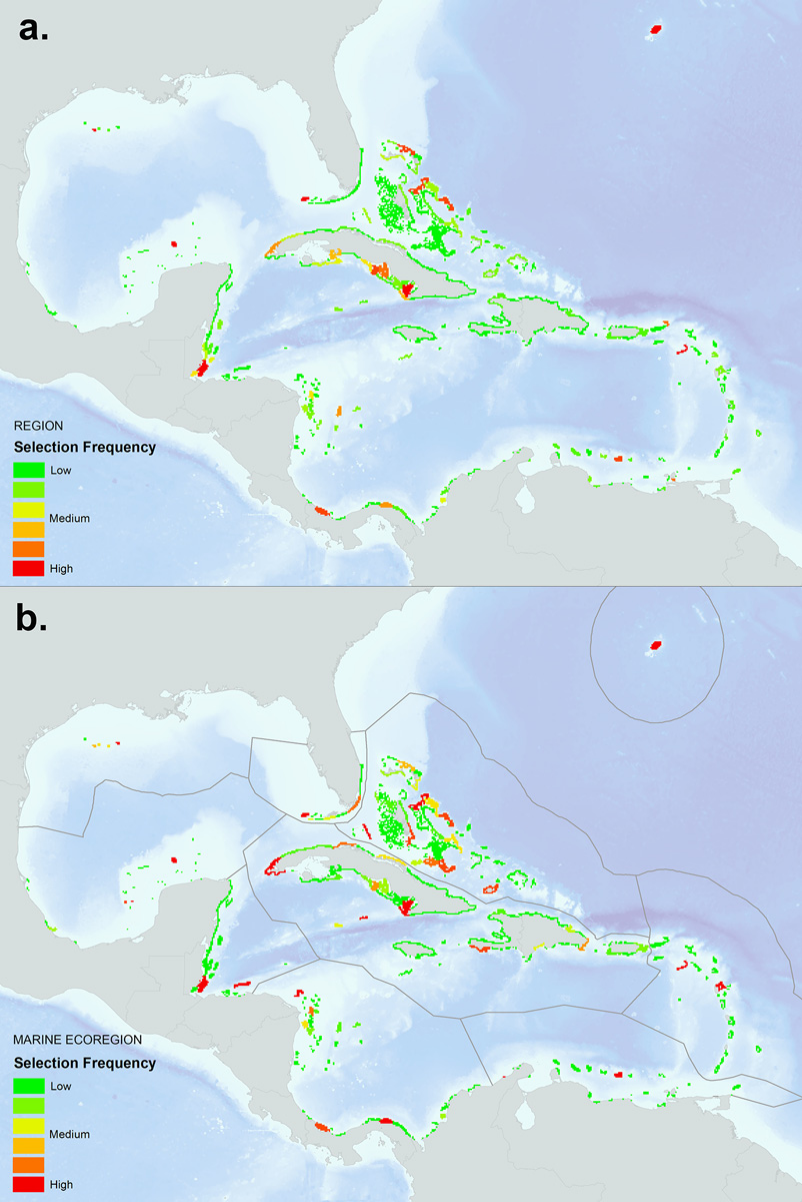
Marxan coral connectivity selection frequency (*i*.*e*. summed solution) that met a 30% target set for local retention and betweenness centrality values by reef unit: a) regional assessment (no strata); and b) stratified by marine ecoregion. These results were based on 100 repetitions using a million iterations per run with a calibrated BLM value of 0.17. A penalty factor of 10 was used and an asymmetric boundary file based on the amount of settled larvae that traveled between reef units. The calculated cost value by reef unit was derived from the Global Map of Human Impacts to Marine Ecosystems [54]. Reef units shaded in red and orange represent those areas that are likely to contribute more to coral reef connectivity.

Figs 8, 9 and 10 show the results of the Marxan analysis comparing the best solutions for the two scenarios: a) targets set using reef area (per reef unit) and a boundary file that uses the Euclidean distance based measure to reef units; and b) a connectivity-based scenario where targets are set using local retention rates and betweenness centrality (per reef unit) and a boundary file that uses the asymmetric strength of connection values calculated between reef units. All targets were met in each of the best solutions presented. Fig 8 shows the results using a 30% target for reef area under two scenarios: (a) no stratification of reef units (*i*.*e*. region as a whole); (b) stratification using ten marine ecoregions. For the first scenario, the solution steers the selection of the 30% target to the reef units with the highest reef area at the lowest cost (based on the marine threat model [54]) using Euclidean distance based measure to reef units as the boundary length. When compared to the no strata results, the marine ecoregion results select 30% of the high reef area-lower cost reef units in each strata, allocating a more representative selection across ecoregions. These solutions can be used as a control to compare the results of the second scenario that integrates the connectivity data for each reef unit and uses the asymmetric strength of connection values calculated between reef units as the boundary length (Fig 9). By comparing these two scenarios, the important reef units that maintain connections in the larval transport model begin to emerge. When no stratification is used (Fig 9B), reef units are selected heavily throughout the core of the network such as the Greater Antilles, including the northern coast of Cuba, Inagua Island in the Bahamas, and areas on the west and east ends of Hispaniola. Interestingly, Little Cayman and Cayman Brac are selected as they represent an important connection bridge, yet no reef units are selected in the Eastern Caribbean perhaps due to the isolated location of these islands. The exception is Bermuda which is characterized by a large reef area and stronger local retention rates. The results of the marine ecoregion stratified runs (Fig 9B) identify the highest connectivity value reef units within each ecoregion based on the model. This solution would likely represent a more resilient design since high connectivity value reef units are selected across the region and not clustered together at the network core. Fig 10 shows the calculated Marxan selection frequency (i.e. summed solution) of the connectivity-based scenario which indicates how many times a reef unit was selected in the algorithm, representing a measure of how important a reef unit is for achieving targets set for the connectivity values. Like Figs 8 and 9, results are shown with (a) no stratification, and (b) stratification by marine ecoregion. These maps can be used to prioritize reef units that consistently contribute to meeting connectivity targets within a regional and marine ecoregional context. Finally, the connectivity results can be overlaid onto existing protected area boundaries to identify weaknesses in the current design in regards to coral reef connectivity.

**Fig 11:**
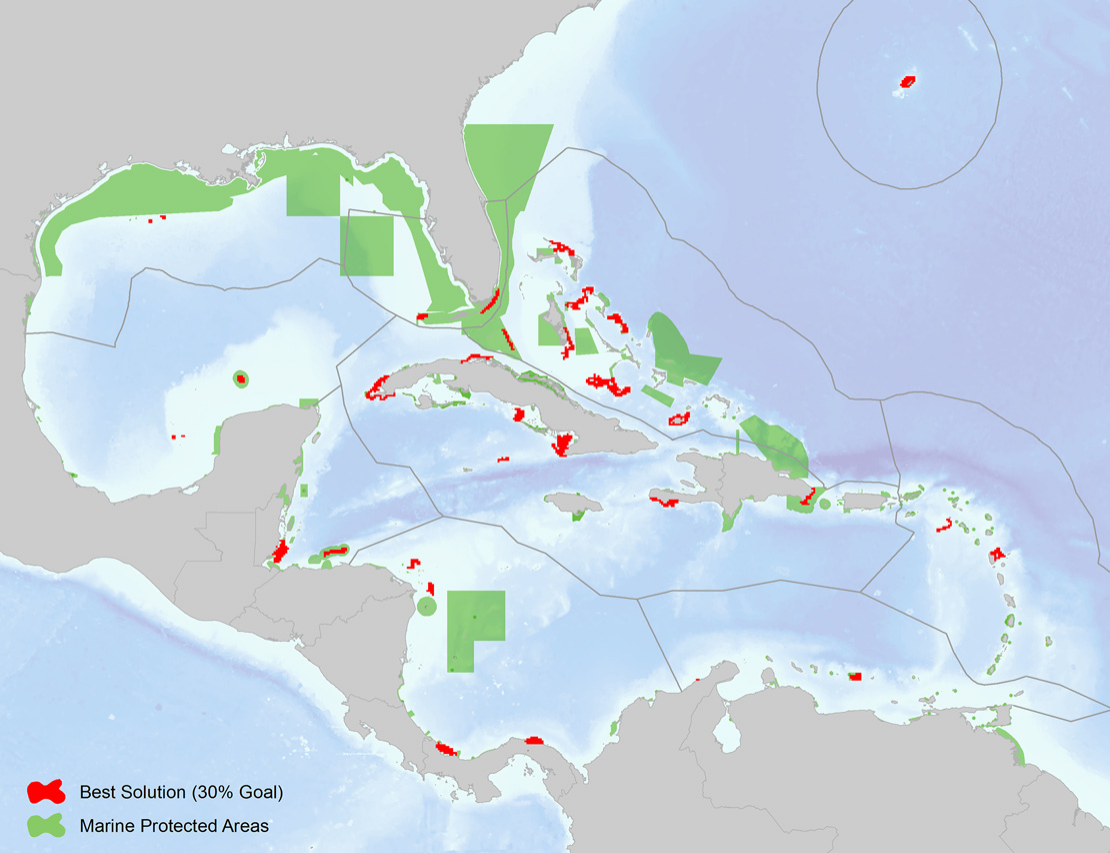
Results of the marine ecoregional coral connectivity best solution (30% target set for local retention and betweenness centrality values by reef unit), overlaid onto the World Database on Protected Areas [56] and The Nature Conservancy’s Marine Protected Area Database of the Insular Caribbean [57].

Our results indicate that of the total coral reef area (16,186 km^2^) mapped within the study area, approximately 6,104 km^2^ (37%) were selected in the Marxan connectivity-based scenario stratified using marine ecoregions. Of these reef areas selected, only 1,424 km^2^ are included within the existing MPA network, indicating that approximately 77% of all coral reefs selected as having a high connectivity value are not included in the existing regional MPA network. We used the current World Database on Protected Areas [56] and The Nature Conservancy’s Marine Protected Area Database of the Insular Caribbean [57] to assess levels of protection, although a vast majority of these parks do not implement management activities (i.e. paper parks). Table 2 shows the breakdown of coral reef area numbers by marine ecoregion and the percentage of selected high value reefs within the existing MPA network. Fig 11 shows the high connectivity value reef units selected by marine ecoregion overlaid onto the current MPA network. Provided that a majority of these high connectivity reef areas are not included in network represents an opportunity for multiple jurisdictions to work collaboratively to expand protection of these critical reef areas, thereby promoting resiliency in the network.

**Table 2:** Breakdown of coral reef area numbers by marine ecoregion and the percentage of selected high value reefs within the existing MPA network.

Marine Ecoregion	Total Coral Reef (km^2^)	Selected as High Connectivity Coral Reef (km^2^)	High Connectivity Reef within MPA Network (km^2^)	% of High Connectivity Reef within MPA Network
Bahamian	2,910.67	1,189.75	6.42	0.54%
Bermuda	739.79	739.77	61.18	8.27%
Eastern Caribbean	920.14	266.98	39.83	14.92%
Floridian	910.05	453.00	212.82	46.98%
Greater Antilles	4,899.78	1,423.91	356.38	25.03%
Northern Gulf of Mexico	226.25	82.01	0.00	0.00%
Southern Caribbean	556.34	156.43	134.48	85.97%
Southern Gulf of Mexico	452.89	336.77	291.74	86.63%
Southwestern Caribbean	2,801.31	867.05	27.80	3.21%
Western Caribbean	1,768.99	588.05	293.43	49.90%
**TOTAL**	**16,186.21**	**6,103.73**	**1,424.08**	**23.33%**

## Discussion

This paper addresses some of the marine connectivity challenges identified in Lagabrielle et al [16]; specifically, integrating connectivity into the design of MPA networks and providing connectivity information that promotes transboundary cooperation and management. Recent conservation assessments in the Caribbean and Gulf of Mexico highlight major gaps in marine protection extent (*i*.*e*. MPA boundaries) and severe deficiencies in management efforts [57,58,59]. Consequently, many countries are taking actions to expand marine protection and bolster management resources. For example, in the Caribbean, several nations and oversea territories have committed to the Caribbean Challenge Initiative to effectively conserve at least 20% of marine habitats by 2020 and putting in place a new sustainable finance architecture that will generate long-term funding for marine and coastal management. With increasing efforts to expand marine protection in the Caribbean Basin and Gulf of Mexico, a unique opportunity exists to incorporate connectivity information to improve and strengthen MPA networks. Marine connectivity modeling has evaded most marine conservation projects due to the high level of sophistication of the model, availability of the data, and the expertise needed to successfully set up, run, and interpret the results [12,22,60]. However, in recent years, there have been enormous improvements in our ability to model ocean currents on spatial and temporal scales that have facilitated progress towards ecosystem-based management. Our results take advantage of consistently mapped coral reefs and recent improvements in oceanographic data and computer simulation programs, to track the potential movement of larvae following a spawning event in a very precise manner, integrating weather and tide information that further increases the accuracy and reliability of these models. This information provides critical insight to coral reef managers seeking to understand how coral reefs are connected throughout the region and can be used as systematic decision support tools for developing a set of regional management strategies that may include establishing new MPAs, protecting specific marine species, and threat abatement for trans-boundary ecosystems.

When comparing the connectivity model results to previous regional models, one can find common areas of strong connections, breaks, and isolations. Roberts [61] was the first to use surface current patterns to map dispersal routes of pelagic larvae and theorize that sites supplied copiously from “upstream” reef areas will be more resilient to recruitment overfishing, less susceptible to species loss, and less reliant on local management than places with little upstream reef. Genetic analysis provides both a valuable tool for measuring genetic structure in marine populations and a means of exploring connectivity predictions with empirical genetic data. Our results follow very similar patters described by Cowen et al [21] finding generally weak larval exchange along the north-western Caribbean tract as described by Taylor and Hellberg [62] and Severance and Karl [63] and a division between the eastern and western Caribbean. The Bahamian Archipelago is also weakly isolated as described by Baums et al. [23]; Taylor and Hellberg [62]; and Vollmer and Palumbi [20] were validated by Foster et al [19] through genetic analysis of two coral species. However, Foster et al [19] had inconclusive results regarding the Mona Passage as a strong genetic barrier as described by Baums et al [23]; Cowen et al [21]; and Taylor and Hellberg [62]. Despite the fact that some connectivity models have been validated using drift buoys and genetic testing [64,65], validation of our connectivity results using genetic analysis is not really appropriate since we are not attempting to model processes such as gene flow, post-settlement, demographics, or mutations.

While the results of this study help to identify general connections between coral reef areas, it is important to note several model limitations such as the treatment of all coral reef areas as equal in their ability to release larvae (proportional to the amount of reef area) when we know that reefs vary in their species composition, condition; and health depending on environmental conditions. Other limitations include the scale at which these ocean circulation models operate (*i*.*e*. 8km cell), which does not take into account local-scale processes, as well as the level of uncertainty in the use of biological parameters. For example, the use of a constant maximum pelagic larval duration (PLD) of 30 days for all coral larvae is applied and may be considered typical for most corals, however, PLD varies between coral species. There also exists uncertainty regarding the scale of larval dispersal and whether populations are mostly self-seeded or are maintained principally by recruits arriving from nearby or even distant reefs [33,46,66]. Our connectivity analysis focuses on larvae of broadcaster corals that “broadcast spawn" into the water to spread offspring. Finally, we used a constant larval mortality rate of 20% when these rates vary by species. Survival of pelagic marine larvae is an important determinant of dispersal potential but few estimates of larval survival are available [67]. Cowen et al [46] suggest that connectivity models often overestimate larval exchange rates because of their inability to adequately account for diffusion and mortality. Jones et al [68] suggest that larval retention and the spatial extent of connectivity in both corals and fish operates independently of larval duration and reef size and is largely influenced by geographic setting Research by Hogan et al [22] highlights the unpredictable nature of connectivity in the real world, and underscores the need for more temporally replicated, empirical measures of connectivity to validate and inform management decisions when using these models. Despite these limitations, our connectivity model is an attempt to address the lack of connectivity data in regional MPA design–integrating the best available data in order to gain insight into regional patterns of coral dispersal and identify important areas to protect from both an ecological and political perspective.

## Conclusions

Despite the many challenges in predicting larval dispersal [69], we demonstrate how a marine connectivity model aids in the identification of important reef connections between coral populations. Urgent action is needed through collaborations by regional governments to design resilient MPA networks that incorporate connectivity information. As stated by Kennedy et al [70], many Caribbean reefs are expected to experience continued structural decline by 2080. In addition, Micheli et al [71] suggest a high vulnerability of Caribbean coral reefs to diversity loss and that protection of multi-species assemblages is needed to maintain ecosystem functions and services. Based on our analysis, only 28% of high connectivity value reefs are included in the current regional MPA network. Our results provide a multi-jurisdictional decision-support tool for coral reef managers who are seeking insight into the behavior of regional coral larval dispersal patterns across the Caribbean Basin and Gulf of Mexico. Based on model output, we identify and prioritize important coral reef linkages zones and spheres of dependence that can be used as a basis for improving coral reef management across multiple jurisdictional boundaries [26]. Modeling the potential direction and magnitude of larval dispersal that is produced across surrounding marine jurisdictions, and integrating these results into a systematic site selection process, serves to guide regional cooperation and promote the collaborative and strategic expansion of marine protected areas aimed at preserving key ecological connections. Berglund et al [72] suggest that connectivity may be more important than habitat quality as selection criterion for MPAs when targeting species with long-distance dispersal; however more research is needed on characterizing and testing predicted dispersal traits for specific species [73]. McCook et al [12] provide a helpful set of ‘rules of thumb’ or practical guidelines that can be applied currently to protect connectivity in marine systems. Innovative new approaches to design MPA networks utilize decision frameworks and can be integrated with model-based connectivity estimates that examine multiple species and scales as well as potential tradeoffs between representation and connectivity [25,74]. These tools are further assisting multi-jurisdictional marine conservation efforts to coordinate policy actions, integrate connectivity information, and make more informed decisions regarding MPA size, spacing, and location. However, given the limitations of connectivity models and the need to consider other aspects of MPA design [10], a portfolio of approaches should be used to protect marine species and habitats, including but not limited to MPAs [14,75]. Ultimately, we hope this study provides guidance on preserving key ecological connections upon which corals depend, but more importantly, incentive that will foster a more coordinated and collaborative regional coral reef management strategy.
